# Obituary: Frank J. Lagerwaard MD, PhD

**DOI:** 10.3389/fonc.2023.1274666

**Published:** 2023-10-24

**Authors:** Enis Özyar, Merav Ben-David

**Affiliations:** ^1^ Acıbadem University, Istanbul, Türkiye; ^2^ Oncology Institute, Assuta Medical Center, Tel-Aviv, Israel; ^3^ Faculty of Health Science, Ben-Gurion University of the Negev, Beer Sheva, Israel

**Keywords:** obituary, Lagerwaard, MR-linac, prostate cancer, pancreas cancer

On July 27, 2023, with the passing of Dr. Frank J Lagerwaard, the profession of radiation oncology lost an outstanding clinician and a brilliant researcher.

Frank spent his childhood years in Rotterdam, Holland. He had graduated from St. Laurens College in 1981 and had earned his medical degree from the Erasmus University in Rotterdam in 1986. For a year between 1989-1990, Frank had worked at Militair Hospitaal Dr. A. Mathijsen as a Radiology Resident and had been a resident of Internal Medicine at the Ignatius Hospital until 1993. He stayed at the Daniel Den Hoed Cancer Center, Rotterdam to complete his radiation oncology training between 1993-2002 and his work resulted in a thesis that earned Lagerwaard a Doctor of Philosophy degree. After 2002, Lagerwaard started to work at the VU University Medical Center (VUMC).

Frank specialized in the treatment of CNS, lung, and pancreatic cancer. After 2006, he was involved in the development of the *MR Linac* program at VUMC. Many scholars are indebted to Lagerwaard for his pioneering work on ablative MR-guided adaptive radiotherapy. He had welcomed many departments within VUMC to share their initial experiences with this cutting edge technology.

Dr. Lagerwaard impact on the field was profound and far-reaching. He was a brilliant researcher, as is evident from the numerous condolences we have received from colleagues all around the world. Each of these contain a personal story of how Frank had positively contributed to their individual careers, professional organizations, and personal lives. His colleagues will always remember this bright, irreverent, kind, and warm-hearted scientist. His contributions and his positive influence on the lives of many will be forever lasting. There can be no nobler legacy.

**Figure:**
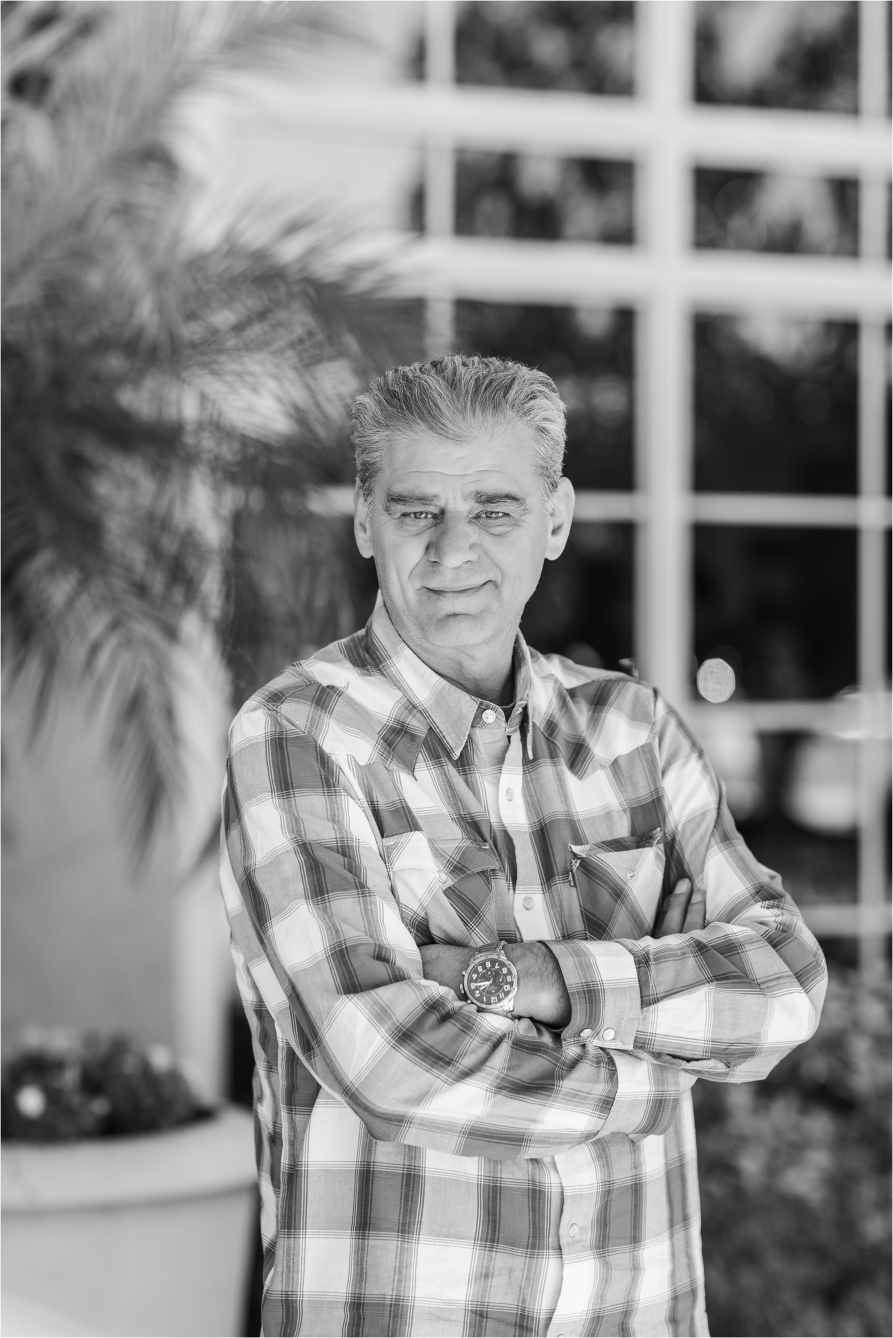


## Author contributions

EO: Writing – original draft, Writing – review & editing. MB-D: Writing – original draft, Writing – review & editing.

